# Precise microbiome engineering using natural and synthetic bacteriophages targeting an artificial bacterial consortium

**DOI:** 10.3389/fmicb.2024.1403903

**Published:** 2024-05-02

**Authors:** Tomoki Tanaka, Ryoga Sugiyama, Yu Sato, Manami Kawaguchi, Kohsuke Honda, Hiroaki Iwaki, Kenji Okano

**Affiliations:** ^1^Department of Chemistry, Materials and Bioengineering, Graduate School of Science and Engineering, Kansai University, Osaka, Japan; ^2^Division of Life Science, Graduate School of Sciences and Technology for Innovation, Yamaguchi University, Yamaguchi, Japan; ^3^Department of Life Science and Biotechnology, Faculty of Chemistry, Materials and Bioengineering, Kansai University, Osaka, Japan; ^4^International Center for Biotechnology, Osaka University, Osaka, Japan; ^5^Industrial Biotechnology Initiative Division, Institute for Open and Transdisciplinary Research Initiatives, Osaka University, Osaka, Japan

**Keywords:** bacteriophage, microbiome engineering, rebooting, synthetic phage, virulent conversion

## Abstract

In natural microbiomes, microorganisms interact with each other and exhibit diverse functions. Microbiome engineering, which enables bacterial knockdown, is a promising method to elucidate the functions of targeted bacteria in microbiomes. However, few methods to selectively kill target microorganisms in the microbiome without affecting the growth of nontarget microorganisms are available. In this study, we focused on the host-specific lytic ability of virulent phages and validated their potency for precise microbiome engineering. In an artificial microbiome consisting of *Escherichia coli*, *Pseudomonas putida*, *Bacillus subtilis*, and *Lactiplantibacillus plantarum*, the addition of bacteriophages infecting their respective host strains specifically reduced the number of these bacteria more than 10^2^ orders. Remarkably, the reduction in target bacteria did not affect the growth of nontarget bacteria, indicating that bacteriophages were effective tools for precise microbiome engineering. Moreover, a virulent derivative of the λ phage was synthesized from prophage DNA in the genome of λ lysogen by *in vivo* DNA assembly and phage-rebooting techniques, and *E. coli*-targeted microbiome engineering was achieved. These results propose a novel approach for precise microbiome engineering using bacteriophages, in which virulent phages are synthesized from prophage DNA in lysogenic strains without isolating phages from environmental samples.

## 1 Introduction

Microorganisms inhabit our entire planet and form very large ecosystems through metabolic and ecological interactions. The collection of microorganisms is called the microbiome. They are distributed across diverse environments including soils, oceans, plants, and animals. For example, tens of trillions of microorganisms live in the human gut (Sender et al., 2016), and gut microbiome composition and microbe-microbe interactions along with microbe-host interactions are closely related to human health. Therefore, an imbalance in or depletion of the gut microbiome causes a variety of diseases, such as inflammatory bowel diseases (Ning et al., 2023), atopic dermatitis (Park et al., 2021), colorectal cancer (Gopalakrishnan et al., 2018), and depression (Guida et al., 2018). Ever-improving DNA sequencing technologies facilitate the comparison of microbial richness and diversity between healthy controls and patients, allowing for the identification of candidate microbes involved in the development and suppression of diseases (Li et al., 2017). However, it is difficult to elucidate the function of a specific microorganism in the presence of a large number of microbes. While gnotobiotic mice colonized with a defined set of microorganisms are powerful tools for examining the function of microorganisms (Atarashi et al., 2011; Tanoue et al., 2019), these models do not represent microbial function in complex microbial communities. The development of a methodology to investigate *in situ* function of microorganisms of interest is thus required.

**Graphical abstract:**
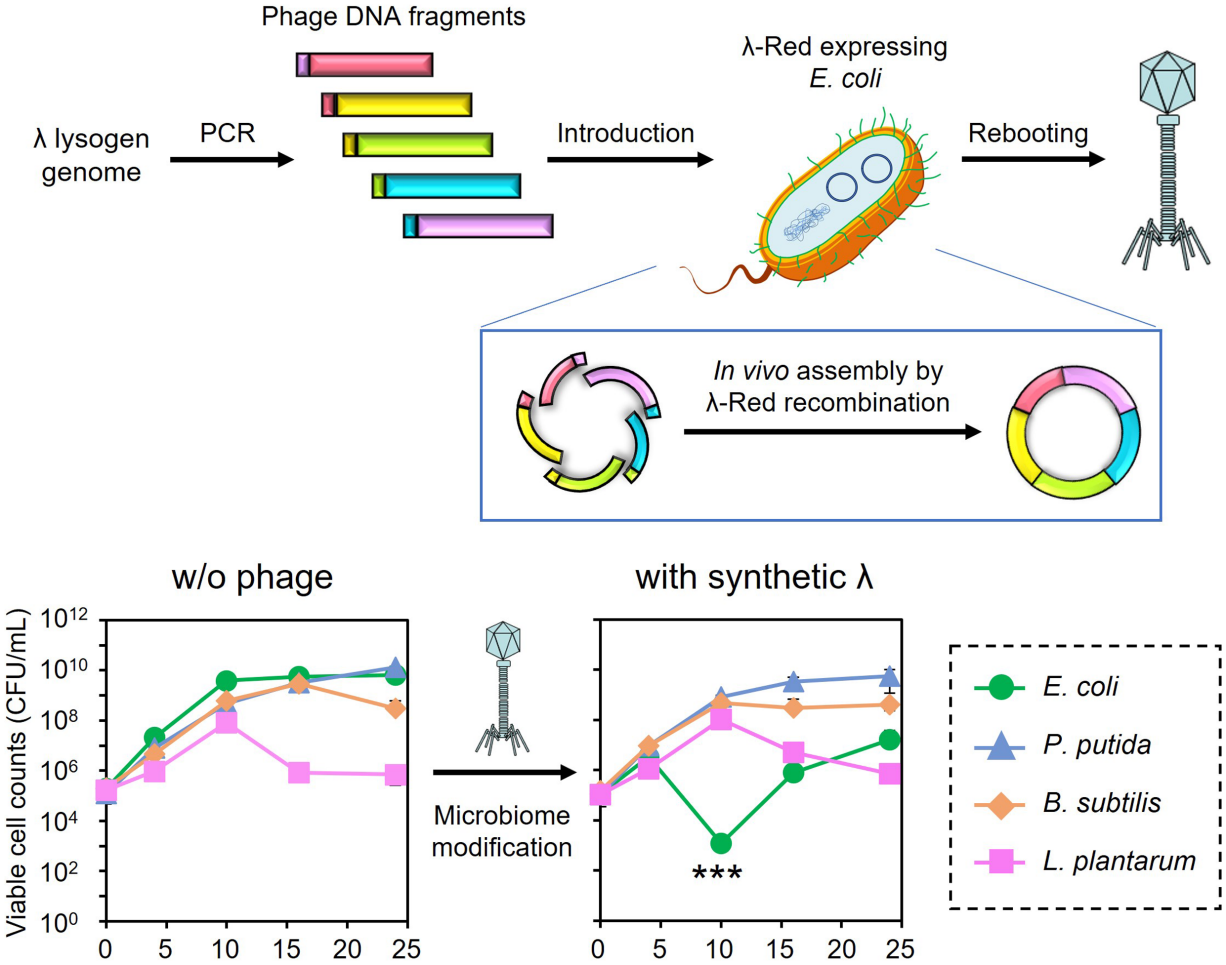


Targeted antimicrobial agents are potential tools to modify the composition of microbiomes in a subtractive manner (Chou et al., 2022). By specifically reducing target microorganisms in microbiomes and evaluating their functional and structural changes, the role of target microorganisms could be elucidated (Hsu et al., 2019; Hizume et al., 2024). Antibiotics are the most frequently used antimicrobial agents. However, they affect numerous bacteria in microbiomes (Patangia et al., 2022) and do not lead to a clear understanding of the functions of specific microorganisms. Some antimicrobial peptides such as bacteriocins show a relatively narrow spectrum compared to antibiotics but still act on a variety of microbes across classes and orders (Rea et al., 2010). To precisely manipulate microbiomes, we previously reported a microbiome engineering technique using peptide nucleic acid (PNA) conjugated with cell-penetrating peptide (CPP) (Hizume et al., 2024). PNA is a DNA analog with a peptide backbone and high biostability. With the aid of CPP, PNA can be delivered into bacterial cells. By designing a sequence of the PNA complementary to the mRNA of the essential gene of the target bacteria, the growth inhibition of target bacteria can be induced. Although PNA selectively inhibited the growth of target bacteria at the species level in the four bacterial coculture system, this can only be achieved when the sequences of the target gene display nucleotide mismatches among species.

Bacteriophages, or simply phages, are prokaryotic viruses infecting and killing bacteria. The initial step of phage infection is the binding of the distal tip of the phage tail to receptors on the bacterial cell surface (Degroux et al., 2023; Klumpp et al., 2023). This step is highly host-specific and phages can recognize their hosts often at the genus and species and sometimes strain levels (Nale et al., 2016; Gao et al., 2018; Fujiki et al., 2022). Virulent phages can produce their replicates inside host cells and they were released from the cells with bacterial lysis, whereas temperate phages occasionally integrate and dormant their genome into the host genome (Sedhom and Solomon, 2023). Considering the characteristics and life cycles of phages, virulent phages could be deployed for subtractive and precise microbiome engineering. Phage therapy refers to the idea of treating specific microorganisms in microbiomes using bacteriophages with the aim of treating microbial infection, and its potential has been demonstrated (Nale et al., 2016). Phage-based microbiome engineering follows the same method but is oriented toward elucidating and controlling microbial function in microbiomes by specifically reducing the numbers of target microorganisms. It has thus become a new research frontier. However, previous studies have evaluated the host specificity of phages against purely cultured bacteria, and while phages can be enriched from complex microbiomes (e.g., sewage) with target bacteria, it is not always clear whether phages are truly host-specific in known complex microbiomes. In addition, as a fundamental issue, microbiome engineering assumes that phages infecting target bacteria are available. Nevertheless, the current phage-isolation method is based on the plaque assay, which requires a pure culture of host bacteria and is time-consuming. Thus, it is not applicable to the isolation of phages infecting uncultured bacteria.

In this study, we achieved subtractive microbiome modification using phages targeting an artificial bacterial consortium consisting of *Escherichia coli*, *Pseudomonas putida*, *Bacillus subtilis*, and *Lactiplantibacillus plantarum*. The phages could selectively kill their target bacteria and did not affect the growth of nontarget bacteria, indicating that phages are effective tools for achieving precise microbiome engineering. Furthermore, an artificial virulent phage was synthesized from the λ lysogen using the phage-rebooting technique, which introduces phage DNA into bacterial cells and makes them synthesize phages. By using the artificial phage, levels of *E. coli* in the artificial microbiome were successfully reduced without a need for phage isolation. These results paved the way for on-demand microbiome engineering and subsequent functional analyses of constituent bacteria in microbiomes.

## 2 Materials and methods

### 2.1 Bacterial strains and media

The bacterial strains used in this study are summarized in Table 1. *E. coli* MG 1655 was obtained from the National Institute of Genetics (Shizuoka, Japan). *P. putida* NBRC 14164 and *B. subtilis* NBRC 111470 were obtained from the Biological Research Center, National Institute of Technology and Evaluation (NITE; Tokyo, Japan). *L. plantarum* NCIMB 8826 was obtained from the National Collection of Industrial, Food and Marine Bacteria (NCIMB; Scotland, UK). They were used as host strains to isolate bacteriophages. A chloramphenicol-resistant *E. coli* MG 1655 derivative (*E. coli* Cm^R^), a neomycin-resistant derivative of *P. putida* NBRC 14164 (*P. putida* Neo^R^) (Hizume et al., 2024), a spontaneous mutant of *B. subtilis* NBRC 111470 with streptomycin resistance (*B. subtilis* Str^R^), and a wild-type *L. plantarum* NCIMB 8826 were used as artificial microbiome components in the microbiome engineering experiments. *B. subtilis* Str^R^ was obtained by directly spreading wild-type *B. subtilis* culture on Luria-Bertani (LB) agar medium with 50 μg/mL streptomycin. After overnight cultivation at 37°C, one of the resulting colonies was picked up and used as a streptomycin-resistant mutant.

**Table 1:** Bacterial strains and bacteriophages used in this study.

Strain or bacteriophage	Relevant description or sequence	Source or reference
Strain		
*Escherichia coli*		
MG 1655	*F^−^* λ*^−^ ilvG^−^ rfb-50 rph-1 lacZ^+^*	National Institute of Genetics
Cm^R^	MG1655 derivative whose *frmA* gene was replaced with the *cat* gene, chloramphenicol resistance	Hizume et al. (2024)
NBRC 3301	λ lysogen whose genome was used as a template for artificial λ synthesis	NITE
DH10B	*F^−^*, *mcrA*, Δ(*mrr*-*hsdRMS*-*mcrBC*), Φ80d*lacZ*ΔM15, Δ*lacX74*, *deoR*, *recA1*, *araD*139, Δ(*ara leu*) 7697, *galU*, *galK*, λ^−^, *rpsL*, *endA1*, *nupG*	Thermo Fisher Scientific
Rosetta2	source of pRARE2	Novagen
*Pseudomonas putida*		
NBRC 14164		NITE
Neo^R^	NBRC 14164 derivative whose *kdsD* gene was replaced with the *kan* gene, neomycin resistance	Hizume et al. (2024)
*Bacillus subtilis*		
NBRC 111470	the same strain with *B. subtilis* 168, *trpC2*	NITE
Str^R^	NBRC 111470 derivative, spontaneous mutant resistant to streptomycin	This study
*Lactiplantibacillus plantarum*		
NCIMB 8826	growth in MRS medium adjusted pH at 6.0	NCIMB
Bacteriophage		
T7	infecting *E. coli*	NITE
λ Δ*int* Δ*cI*	synthetic λ lacking *int* and *cI* genes	This study
ΦPpMK2-1	isolated from the soil, infecting *P. putida*	This study
ΦBsKO1-1	isolated from the soil, infecting *B. subtilis*	This study
ΦLpTT2	isolated from the wastewater, infecting *L. plantarum*	This study

*E. coli*, *P. putida*, and *B. subtilis* strains were cultivated in LB medium, while *L. plantarum* was cultivated in de Man-Rogosa-Sharpe (MRS) broth (Difco Laboratories, Detroit, MI, USA). For solid media, 1.5% (w/v) agar was added. If necessary, 30 μg/mL chloramphenicol, 50 μg/mL neomycin, and 50 μg/mL streptomycin were added to the media. *E. coli*, *B. subtilis*, and *L. plantarum* were routinely cultivated at 37°C, while *P. putida* was cultivated at 30°C, unless specified otherwise. Glycerol stocks of bacterial cultures were prepared for *E. coli* Cm^R^, *P. putida* Neo^R^, *B. subtilis* Str^R^, and *L. plantarum*. Each bacterium was cultivated for 12 h and 180 rpm. Then, 40 μL of each bacterial culture was mixed with an equal volume of 30% (w/v) glycerol solution and stored at −80°C until use.

*E. coli* DH10B (Thermo Fisher Scientific, Tokyo, Japan) harboring pCas and pRARE2 was used as a host for *in vivo* assembly and for rebooting the synthetic phage genome. pCas was a gift from Sheng Yang (Addgene plasmid #62225^1^ RRID: Addgene_62225) (Jiang et al., 2015), allowing the expression of λ-Red recombinase genes (Datsenko and Wanner, 2000). pRARE2 was extracted from *E. coli* Rosetta2 (Novagen, Madison, WI, USA) and used for supplying the seven tRNAs that are uncommon in *E. coli*.

### 2.2 Bacteriophages

The bacteriophages used in this study are also summarized in Table 1. The T7 phage was obtained from the Biological Research Center, NITE. The bacteriophages infecting *P. putida* or *B. subtilis* were isolated from soil samples collected from the Kansai University campus. Approximately 5 g of the soil samples was mixed with 45 mL of sterilized deionized water and centrifuged at 4,000 × *g* for 5 min. The supernatant was filtered through a 0.22 μm pore-size syringe filter (PES025022S; Membrane Solutions, Auburn, WA, USA) to remove bacterial cells. Then, the filtrate was concentrated 50 times by ultrafiltration using an Amicon® Ultra-15 centrifugal filter device (Merck Millipore; Darmstadt, German) and used for phage screening. The bacteriophage infecting *L. plantarum* was isolated from a sewage sample obtained from a sewage treatment plant in the Osaka Prefecture. Approximately 360 mL of the sewage sample was centrifuged at 4,000 × *g* at 4°C for 60 min, and the supernatant was collected. NaCl and polyethylene glycol 8,000 were then added to the supernatant at final concentrations of 5% (w/v) and 10% (w/v), respectively. After incubating at 4°C overnight, the sample was centrifuged at 4,000 × *g* at 4°C for 90 min, and the supernatant was removed. The resulting precipitate was suspended in 1 mL of SM buffer (NaCl, 5.8 g/L; MgSO_4_ 7H_2_O, 2.0 g/L; gelatin, 0.1 g/L; Tris–HCl (pH 7.5), 50 mM) and impurities were removed by filtration through a 0.22 μm filter (Membrane Solutions). The resulting filtrate was used for phage screening. Bacteriophage isolation was performed by the double agar overlay method (Karaynir et al., 2023) with some modifications. Briefly, 100 μL overnight culture of each host strain was mixed with the equivalent volume of each pretreated environmental sample. The mixture was added to 4 mL of 0.5% (w/v) soft agar medium pre-warmed at 48°C and overlaid on 1.5% (w/v) agar medium. After overnight cultivation, a single plaque was suspended in SM buffer, and phage isolation was repeated thrice to obtain single bacteriophages. The purified bacteriophages infecting *P. putida*, *B. subtilis*, and *L. plantarum* were designated as ΦPpMK2-1, ΦBsKO1-1, and ΦLpTT2, respectively. Genome sequencing and characterization of these phages were conducted as described in the Materials and Methods section of the Supplementary material. The titer of each phage suspension, including the T7 phage suspension, was determined as plaque forming units (PFU) per milliliter in the plaque assay by the double agar overlay method.

### 2.3 Examination of dose dependency of the phages for inhibiting the growth of host bacteria

Glycerol stocks of *E. coli* Cm^R^, *P. putida* Neo^R^, and *B. subtilis* Str^R^ were inoculated in 4 mL LB broth and that of *L. plantarum* was inoculated in 4 mL MRS broth. They were cultivated for 12 h and bacterial cells were collected by centrifuging at 8,000 × *g* at 4°C for 1 min. The resulting pellets were washed twice by 2 mM PIPES-NaOH (pH 6.8) and resuspended in the same buffer. Each of the four bacterial cells was inoculated in LB broth supplemented with 0.5% (w/v) CaCO_3_ to reach an initial cell concentration of 10^5^ colony-forming units (CFUs) per milliliter. Then, the phage suspensions were added to the culture of their susceptible host bacteria at multiplicity of infection (MOI) values of 0.001 and 0.01. For an evaluation of the dose dependency of ΦLpTT2, the phage was also added at MOI values of 0.1 and 1. After 10 h of cultivation at 34°C and 180 rpm, the cultures were serially diluted with PIPES-NaOH (pH 6.8), and 50 μL of the dilutants were spotted on the agar media. After overnight cultivation, the numbers of colonies were counted and viable cell counts were determined.

### 2.4 Phage specificity assessment

The growth inhibitory effect of the bacteriophages against the nonhost bacteria was also assessed. Bacterial cultures were prepared as described above and the phage suspensions of T7 phage, ΦPpMK2-1, ΦBsKO1-1, and ΦLpTT2 were added to cultures of the nonhost bacteria at MOI values of 0.001, 0.01, 0.001, and 1, respectively. After 10 h of cultivation at 34°C and 180 rpm, the numbers of viable cells were determined as described above.

### 2.5 Microbiome engineering using bacteriophages

Cell suspensions of *E. coli* Cm^R^, *P. putida* Neo^R^, *B. subtilis* Str^R^, and *L. plantarum* were prepared as described above. They were co-inoculated in 4 mL LB broth with 0.5% (w/v) CaCO_3_ to reach 10^5^ CFU/mL of each. Then, each of the phages was added to the culture at the MOI values described above. The artificial microbiome was cultivated at 34°C and 180 rpm, and the cultures were collected at 0, 4, 10, 16, and 24 h. Then, 50 μL of dilutants were spotted on the four different agar media; LB medium supplemented with chloramphenicol, neomycin, or streptomycin, and MRS medium prepared at pH 6.0. These media allow the individual measurement of viable cell numbers of *E. coli* Cm^R^, *P. putida* Neo^R^, *B. subtilis* Str^R^, and *L. plantarum* in the artificial microbiome.

### 2.6 Construction and rebooting of the synthetic phage DNA from the prophage DNA in the λ lysogen

The synthetic λ phage genome, whose lysogeny-related genes are deleted, was constructed and rebooted as follows. Genomic DNA was extracted from a λ lysogen *E. coli* NBRC 3301. The prophage region in the genome (Genbank, BJLE01000002.1), except for the regions including *attL* and *int* (position 1,680,514–1,681,672), *cI* (position 1,690,017–1,690,730), and *attR* (position 1,729,017–1,729,031) were split into five regions. Each region was amplified by PCR from the genome of *E. coli* NBRC 3301 using the primers listed in Supplementary Table S1. PCR was performed using the KOD One PCR Master Mix (TOYOBO CO., Ltd., Osaka, Japan). The primers were designed to create an overlap region of 47–50 bp with the adjacent fragments. The amplified fragments were subjected to agarose gel electrophoresis and purified with a FastGene Gel/PCR Extraction Kit (Nippon Genetics, Tokyo, Japan) according to the supplier’s instructions. The DNA concentrations of each fragment were measured using the 1× dsDNA HS Assay Kit on a Qubit Flex Fluorometer (Thermo Fisher Scientific).

The *in vivo* assembly and rebooting of the synthetic λ phage genome were performed according to the method by Cheng et al. (2022) with some modifications. *E. coli* DH10B/pCas/pRARE2 was cultivated at 30°C in SOB medium (Difco Laboratories) supplemented with 17 μg/mL chloramphenicol, 50 μg/mL kanamycin, and 10 mM arabinose for the induction of λ-Red recombinase. Once the OD_600_ reached 0.5, competent cells were prepared as described by Green and Rogers (2013). Approximately 60 fmoles of each phage DNA fragment were added to the 200 μL of competent cells and incubated at 42°C for 2 min. Then, the cells were immediately transferred to ice. After 3 min of incubation, 1 mL of pre-warmed LB medium at 37°C was added, followed by cultivation at 37°C and 180 rpm for 12 h to induce λ-Red recombinase-mediated DNA assembly and rebooting of the resulting phage genome. The synthesized phage particles were separated from the host cells by 10% chloroform treatment, followed by centrifugation at 6,000 × g for 5 min and filtration with a 0.22 μm pore-size syringe filter (PES025022S; Membrane Solutions). Then, 100 μL of the filtrate was mixed with an overnight culture of *E. coli* MG1655 and incubated at 37°C and 180 rpm for 5 h. The mixture was then subjected to the double agar overlay method to obtain plaques. The accuracy of gene assembly was validated by PCR amplification of the 600-bp joint regions of each fragment directly from a plaque of the synthetic phage and following DNA sequencing analyses. The synthesized phage was designated λ Δ*int* Δ*cI*.

## 3 Results

### 3.1 Isolation and characterization of the bacteriophages

To evaluate the applicability of the bacteriophages to precise microbiome engineering, the subtractive modification of an artificial bacterial consortium consisting of *E. coli*, *P. putida*, *B. subtilis*, and *L. plantarum* was performed in this study. This bacterial combination was chosen because it includes Gram-negative and Gram-positive bacteria from different genera. This combination also allows the measurement of viable cell counts of individual microorganisms using selective media because antibiotics-resistant derivatives were available for *E. coli*, *P. putida*, and *B. subtilis* (see the Materials and methods section), and *L. plantarum* has acid tolerance. In addition, the four bacterial species do not affect the growth of other species under the experimental conditions. Therefore, this model is suitable for testing the specificity of the phages in the microbiome rather than in pure culture systems. The phages infecting *P. putida* NBRC 14164 and *B. subtilis* NBRC 11470 were isolated from the soil extract, whereas the phage infecting *L. plantarum* NCIMB 8826 was isolated from a wastewater treatment plant’s wastewater. The presence of the phages was confirmed by the formation of plaques on the agar media (Supplementary Figure S1). The purified phages from single plaques were designated ΦPpMK2-1, ΦBsKO1-1, and ΦLpTT2, respectively. All phages formed clear plaques, suggesting that the phages were virulent phages. The genomes of the isolated phages were sequenced with a MinION sequencer, and their taxonomic placement was determined (Table 2). All phages belong to the class *Caudoviricetes* and phages in this class have icosahedral head and tail fibers. Phage infection is triggered by the specific binding of the distal end of phage tails to receptor on the cell surface of host cells (Zinke et al., 2022). The genome size o*f* ΦPpMK2-1 was 38,794 bp with GC contents of 57.4% and 54 genes. Its genome sequence showed the highest similarity (92.4%) with *Pseudomonas* phage 17A and was found to belong to the genus *Ghunavirus*. The genome sequence of ΦBsKO1-1 (142,728 bp, GC content = 39.9%, number of genes = 285) showed the highest similarity (79.6%) with *Bacillus* phage CampHawk and was found to belong to the genus *Okubovirus*. Conversely, the genome sequence of ΦLpTT2 (37,440 bp, GC content = 45.3%, number of genes = 71) had low homology to known viral genomes and showed 62.5% similarity with *Pediococcus* phage cIP1, which belongs to the class *Caudoviricetes*. The genomes of *Pseudomonas* phage 17A (NCBI RefSeq, NC_048201.1), *Bacillus* phage CampHawk (GenBank, KF669649.1), and *Pediococcus* phage cIP1 (GenBank, JN051154.1) were predicted to have no lysogeny-related genes such as integrase and repressor. This further emphasizes that ΦPpMK2-1, ΦBsKO1-1, and ΦLpTT2 are virulent phages.

**Table 2:** Genome information of the isolated bacteriophages.

Genome information	ΦPpMK2-1	ΦBsKO1-1	ΦLpTT2
Length (bp)	38,794	142,728	37,440
GC contents (%)	57.4	39.9	45.3
No. of genes	54	285	71
Coverage (×)	148	26	37
Circular	No	No	Yes
Closest species	*Pseudomonas* phage 17A	*Bacillus* phage CampHawk	*Pediococcus* phage cIP1
Mean identity (%)	92.4	79.6	63.4
Class	*Caudoviricetes*	*Caudoviricetes*	*Caudoviricetes*
Order	None	None	None
Family	*Autographiviridae*	*Herelleviridae*	None
Subfamily	*Studiervirinae*	*Spounavirinae*	None
Genus	*Ghunavirus*	*Okubovirus*	None
Accession no.	LC805023	LC805024	LC805022

### 3.2 Examination of the dose dependency of the phages for inhibiting host bacteria growth

**Figure 1:**
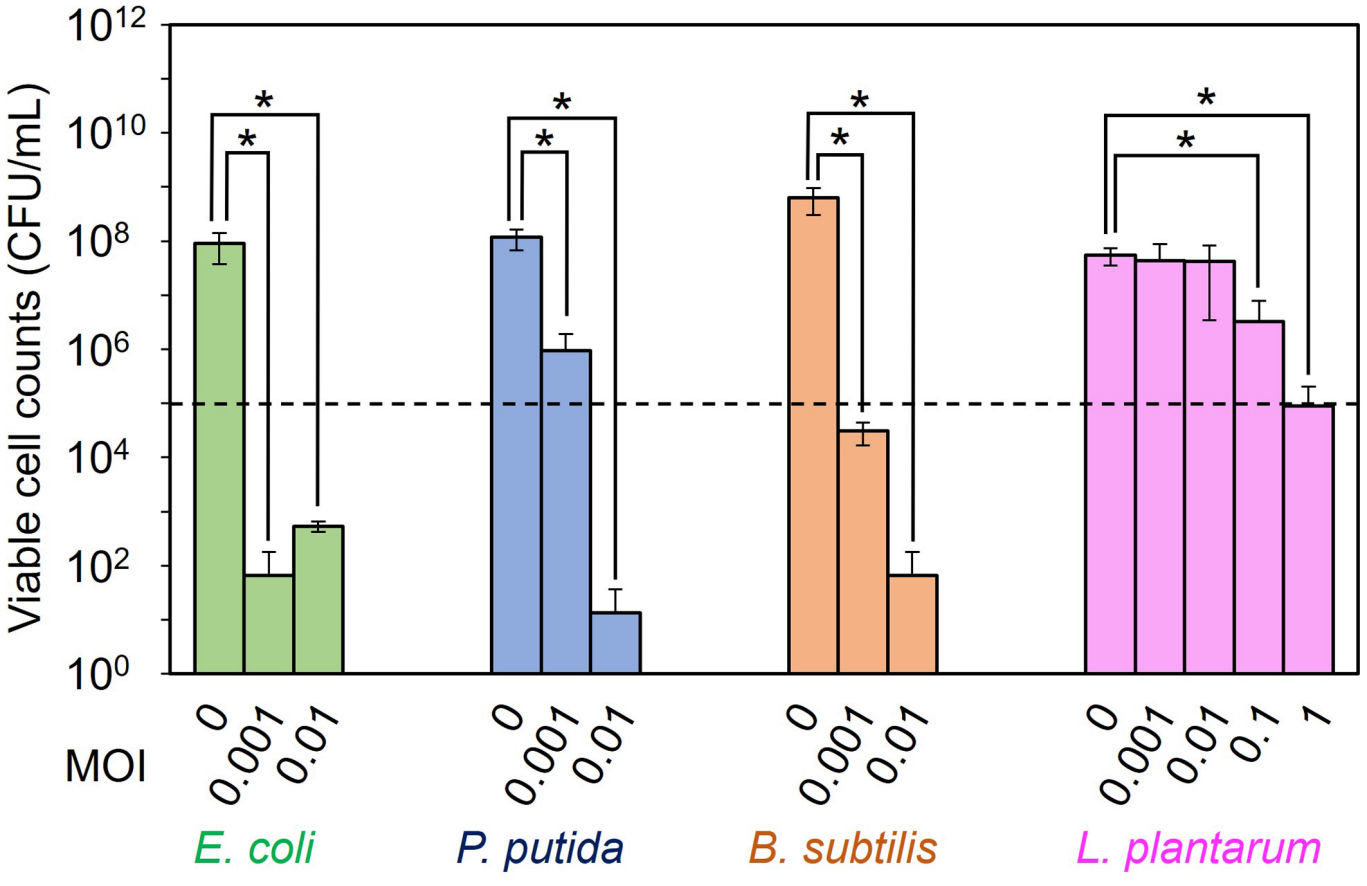
Examination of the dose dependency of the phages for inhibiting the growth of the host bacterium. T7 phage, ΦPpMK2-1, ΦBsOK1-1, and ΦLpTT2 were added to the cultures of their respective host bacterium, *E. coli*, *P. putida*, *B. subtilis*, and *L. plantarum* at different multiplicity of infection (MOI) values. After 10 h of cultivation, the viable cell counts of each bacterium were measured. The dotted line represents the initial viable cell counts (1.00 × 10^5^ CFU/mL). Data bars show the mean ± standard deviation of three independent experiments. The viable cell counts of each bacterium with its respective bacteriophage were compared to those in the no-phage conditions. Asterisks indicate *p* values less than 0.05 in the *t*-test.

The dose dependency of T7, ΦPpMK2-1, ΦBsKO1-1, and ΦLpTT2 to inhibit the growth of their respective host strain (*E. coli*, *P. putida*, *B. subtilis*, and *L. plantarum*, respectively) was examined in monoculture systems (Figure 1). When *E. coli* Cm^R^, *P. putida* Neo^R^, *B.subtilis* Str^R^, and *L. plantarum* were cultivated for 10 h without phage infection, the viable cell numbers increased from an initial cell concentration of 1.00 × 10^5^ CFU/mL to 9.00 × 10^7^, 1.17 × 10^8^, 6.28 × 10^8^, and 5.47 × 10^7^ CFU/mL, respectively. Alternatively, the viable cell numbers were reduced by phage infection in all tested conditions. Infection by T7 phage at MOI values of 0.001 and 0.01 reduced the viable cell numbers of *E. coli* Cm^R^ to 6.67 × 10^1^ CFU/mL and 5.33 × 10^2^ CFU/mL, respectively. This result confirmed that the addition of 10^2^ PFU/mL (MOI = 0.001) of T7 phage was enough to decrease the viable cell numbers from the initial cell concentration of 1.00 × 10^5^ CFU/mL. Following this criterion, the addition of ΦPpMK2-1 at MOI of 0.001 was insufficient to decrease the number of viable cells of *P. putida* Neo^R^ from the initial level, as the viable cell numbers of *P. putida* were 9.56 × 10^5^ CFU/mL. The complete growth inhibition of *P. putida* was achieved by the addition of ΦPpMK2-1 at 10^3^ PFU/mL (MOI = 0.01), reducing the viable cell numbers to 1.33 × 10^1^ CFU/mL. For ΦBsKO1-1, the addition of phage at 10^2^ PFU/mL (MOI = 0.001) was enough to inhibit the growth of *B. subtilis* Str^R^ (3.08 × 10^4^ CFU/mL). Unfortunately, the addition of ΦLpTT2 to the culture of *L. plantarum* at MOI values of 0.001 and 0.01 was insufficient to inhibit the growth of *L. plantarum*. The viable cell numbers of *L. plantarum* were 4.40 × 10^7^ CFU/mL and 4.27 × 10^7^ CFU/mL, respectively, which are comparable to that in the nontreated condition (5.47 × 10^7^ CFU/mL). Accordingly, the dose dependency of ΦLpTT2 was examined at higher MOI, and the addition of 10^5^ PFU/mL of ΦLpTT2 (MOI = 1) could inhibit the growth of *L. plantarum*. The viable cell number was 8.97 × 10^4^ CFU/mL. From these results, the titers for inhibiting the host growth of T7, ΦPpMK2-1, ΦBsKO1-1, and ΦLpTT2 were determined as 10^2^, 10^3^, 10^2^, and 10^5^ PFU/mL (MOI = 0.001, 0.01, 0.001, and 1), respectively, and applied in subsequent experiments.

### 3.3 Assessment of host specificity of the bacteriophages

**Figure 2:**
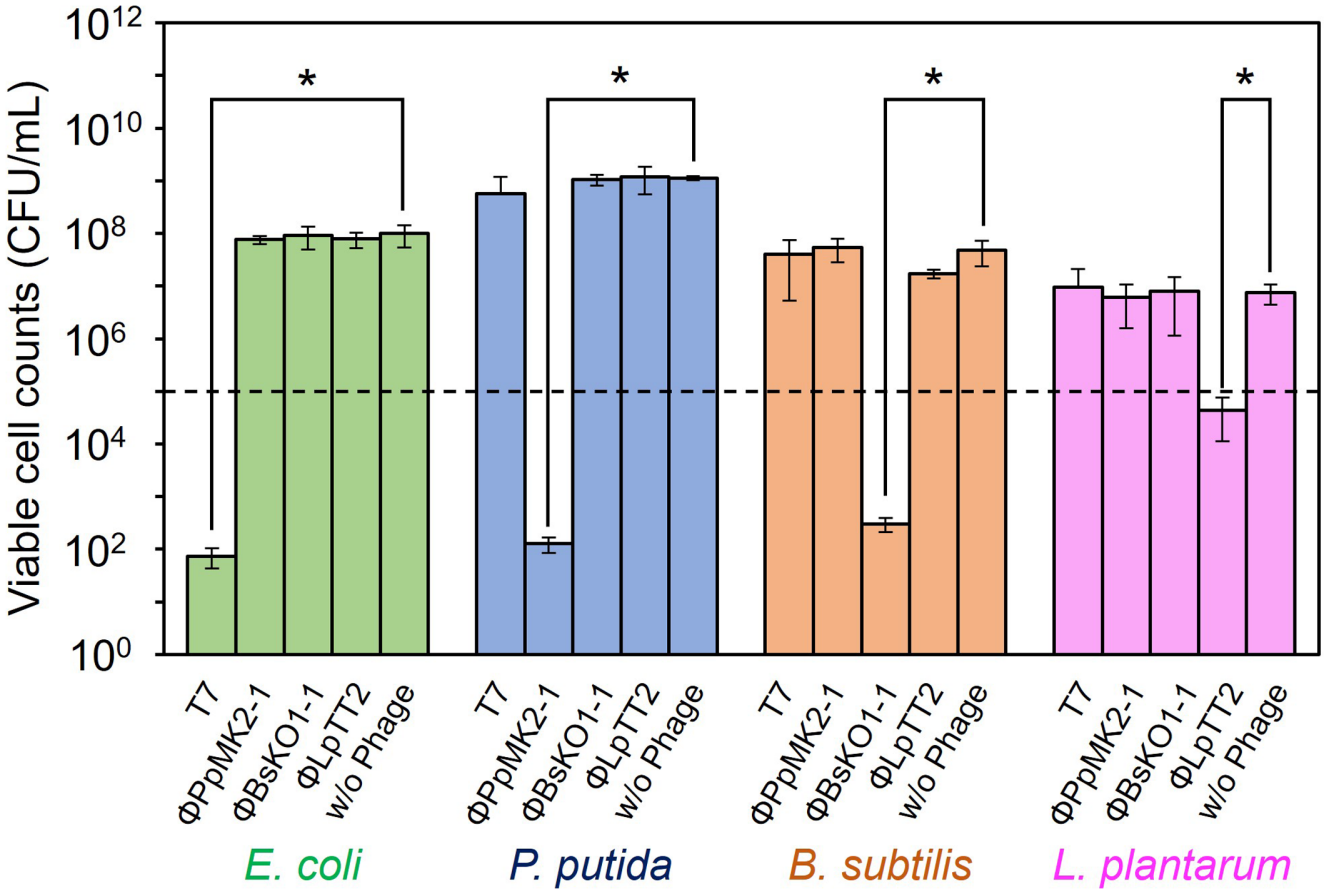
Evaluation of the host specificity of the bacteriophages. Each of the four phages was added to the culture of *E. coli*, *P. putida*, *B. subtilis*, and *L. plantarum*. T7 and ΦBsOK1-1 phages were added to the bacterial cultures at MOI of 0.001, whereas ΦPpMK2-1 and ΦLpTT2 were added at MOI values of 0.01 and 1, respectively. The dotted line represents the initial viable cell counts (1.00 × 10^5^ CFU/mL). Data bars show the mean ± standard deviation of three independent experiments. For statistical analyses, the viable cell counts of each bacterium with and without the addition of the bacteriophage indicated were compared. Asterisks indicate *p* values less than 0.05 in the *t*-test.

The specificity of the phages to the host bacterium was evaluated to ensure that each phage did not affect the growth of the nonhost bacteria (Figure 2). We defined the criteria for determining whether each phage is specific against the target bacterium as satisfying the following two conditions: (1) phage can reduce the viable cell counts of target bacterium below an initial viable cell counts (10^5^ CFU/mL) after 10 h cultivation. (2) Phage does not reduce the viable cell counts of nontarget bacteria compared to those without phage with a statistical significance. When T7 phage was added to the culture of *E. coli* Cm^R^, the viable cell number was 7.33 × 10^1^ CFU/mL, which was significantly lower than those without phage (9.93 × 10^7^ CFU/mL: *p* = 0.031). Contrarily, the viable cell numbers of *P. putida* Neo^R^, *B. subtilis* Str^R^, and *L. plantarum* incubated with T7 phage were 5.72 × 10^8^, 4.00 × 10^7^, and 9.59 × 10^6^ CFU/mL (*p* = 0.17, 0.25, and 0.37), respectively. Therefore, the T7 phage reduced only the viable cell number of *E. coli* and did not affect the growth of the other three bacteria. ΦPpMK2-1 also reduced only the viable cell number of its original host, *P. putida*, which was reduced to 1.27 × 10^2^ CFU/mL (*p* = 0.031), while no significant reduction was observed in that of *E. coli*, *B. subtilis*, and *L. plantarum* (*p* = 0.19, 0.091, and 0.27, respectively). Similarly, ΦBsKO1-1 and ΦLpTT2 were effective only against their native hosts. The addition of ΦBsKO1-1 and ΦLpTT2 reduced only the viable cell numbers of *B. subtilis* and *L. plantarum*, respectively, achieving statistical significance (*p* = 0.038 and 0.025, respectively). These results indicated that all four phages were highly specific for their host bacterium in pure culture systems.

### 3.4 Subtractive modification of the artificial microbiome using the bacteriophages

**Figure 3:**
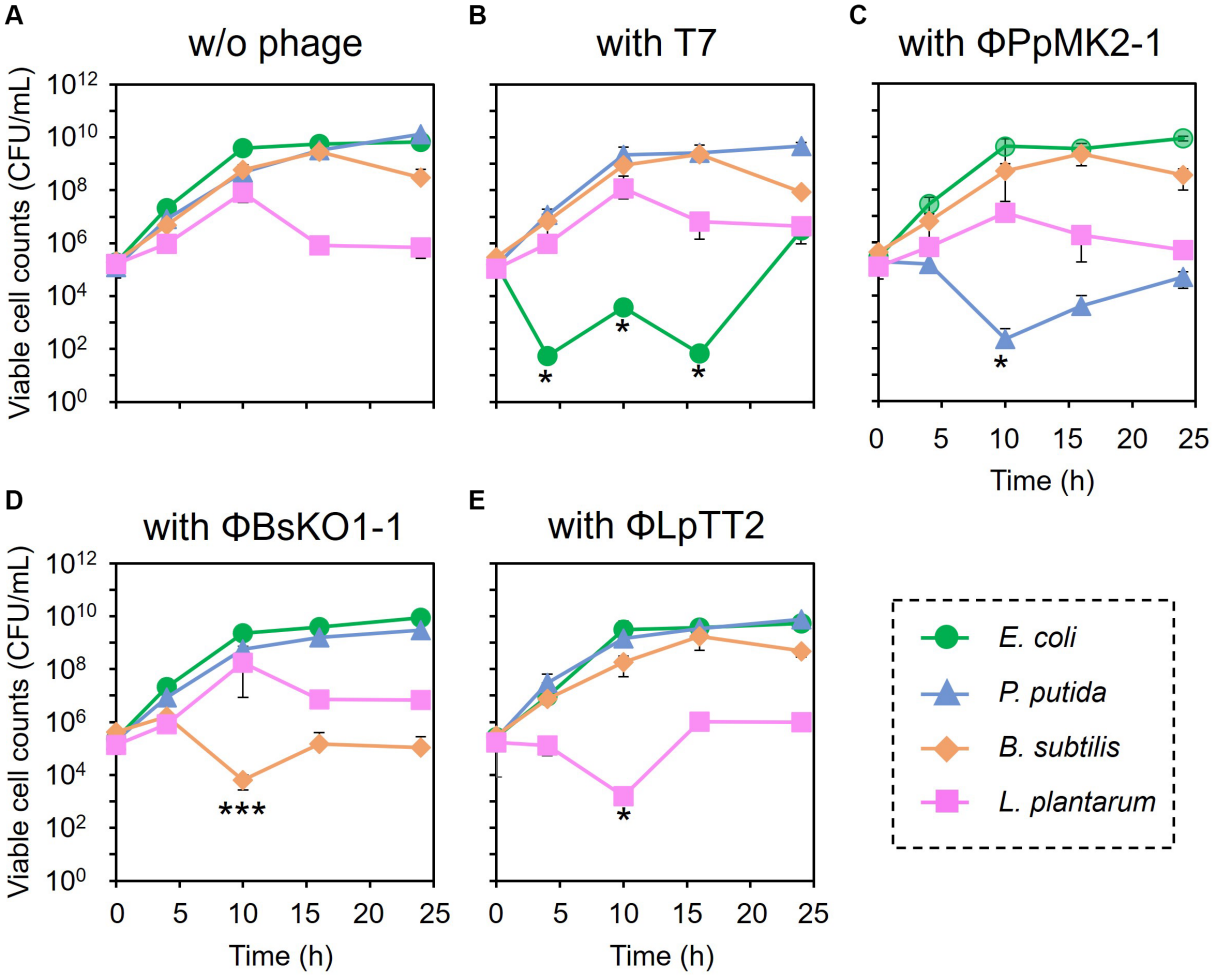
Microbiome engineering of the artificial microbiome by using bacteriophages. *E. coli* (green circles), *P. putida* (blue triangles), *B. subtilis* (brown diamonds), and *L. plantarum* (pink squares) were cocultivated for 24 h without phage **(A)**, with T7 phage at MOI 0.001 **(B)**, with ΦPpMK2-1 at MOI 0.01 **(C)**, with ΦBsOK1-1 at MOI 0.001 **(D)**, and with ΦLpTT2 at MOI 1 **(E)**. The samples were collected at 0, 4, 10, 16, and 24 h, and the viable cell numbers were determined. Data points show the mean ± standard deviation of three independent experiments. For statistical analyses, the viable cell counts of the target bacteria with their respective bacteriophages were compared with their initial cell concentrations. Asterisks and triple asterisks indicate *p* values less than 0.05 and 0.005 in the *t*-test.

The feasibility of microbiome engineering using bacteriophages was tested in an artificial microbiome consisting of four bacterial species. When no phage was added to the microbiome, the viable cell numbers at 24 h were 6.67 × 10^9^ CFU/mL for *E. coli* Cm^R^, 1.33 × 10^10^ CFU/mL for *P. putida* Neo^R^, 3.03 × 10^8^ CFU/mL for *B. subtilis* Str^R^, and 7.01 × 10^5^ CFU/mL for *L. plantarum* (Figure 3A). As expected, the addition of the bacteriophages significantly reduced the viable cell numbers of their respective host bacterium from the initial concentration of 1.00 × 10^5^ CFU/mL (Figures 3B–E). Their cell numbers decreased to 5.33 × 10^1^ CFU/mL at 4 h for *E. coli* (*p* = 0.034), 2.27 × 10^2^ at 10 h for *P. putida* (*p* = 0.045), 6.27 × 10^3^ at 10 h for *B. subtilis* (*p* = 0.0034), and 1.60 × 10^3^ CFU/mL at 10 h for *L. plantarum* (*p* = 0.035). In particular, the viable cell counts of *E. coli* were low even after 10 and 16 h of cultivation, with numbers of 3.74 × 10^3^ (*p* = 0.033) and 6.67 × 10^1^ CFU/mL (*p* = 0.034), respectively.

**Figure 4:**
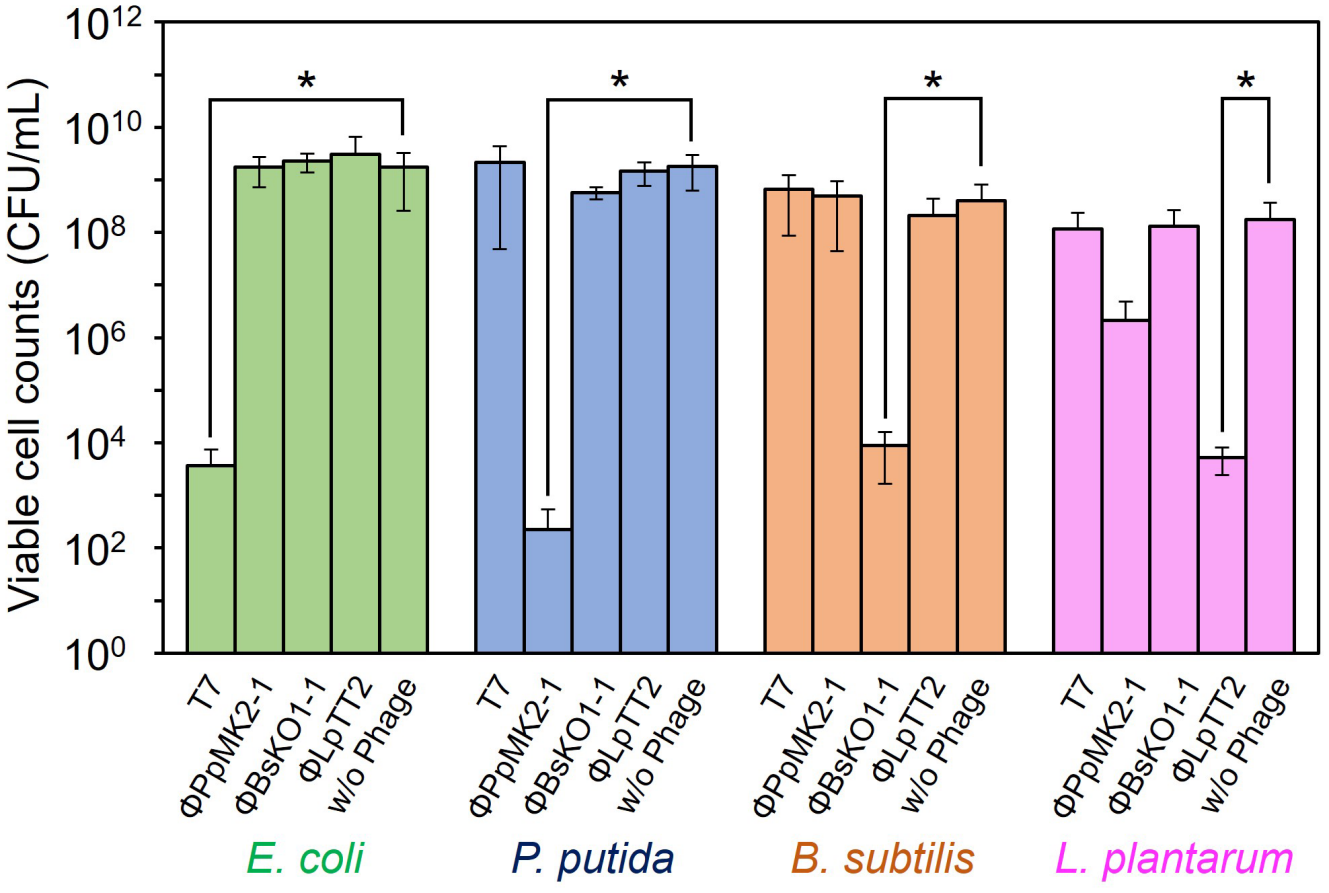
Evaluation of the host specificity of the bacteriophages in the coculture system. The viable cell counts of *E. coli* (green bars), *P. putida* (blue bars), *B. subtilis* (brown bars), and *L. plantarum* (pink bars) at 10 h with and without the addition of the bacteriophage indicated were compared. Data bars show the mean ± standard deviation of three independent experiments. Asterisks indicate *p* values less than 0.05 in the *t*-test.

In contrast, none of the phages affected the growth of the nonhost bacteria in the coculture system (Figure 4). When T7 phage was added to the artificial microbiome, the viable cell numbers of *P. putida*, *B. subtilis*, and *L. plantarum* were 2.17 × 10^9^, 8.80 × 10^8^, and 1.17 × 10^8^ CFU/mL, respectively. These values were almost the same as those in the nonphage conditions (4.67 × 10^8^, 6.01 × 10^8^, and 8.00 × 10^7^ CFU/mL) with *p* values of 0.15, 0.084, and 0.36, respectively. Similarly, the addition of ΦPpMK2-1, ΦBsKO1-1, and ΦLpTT2 did not interfere with the growth of the nonhost bacteria. These results demonstrate that the high host specificity of the phages is also exhibited in a complex culture system, suggesting that microbiome engineering by using bacteriophages is possible.

### 3.5 Subtractive modification of the artificial microbiome using the synthetic λ Δ*int* Δ*cI* phage

**Figure 5:**
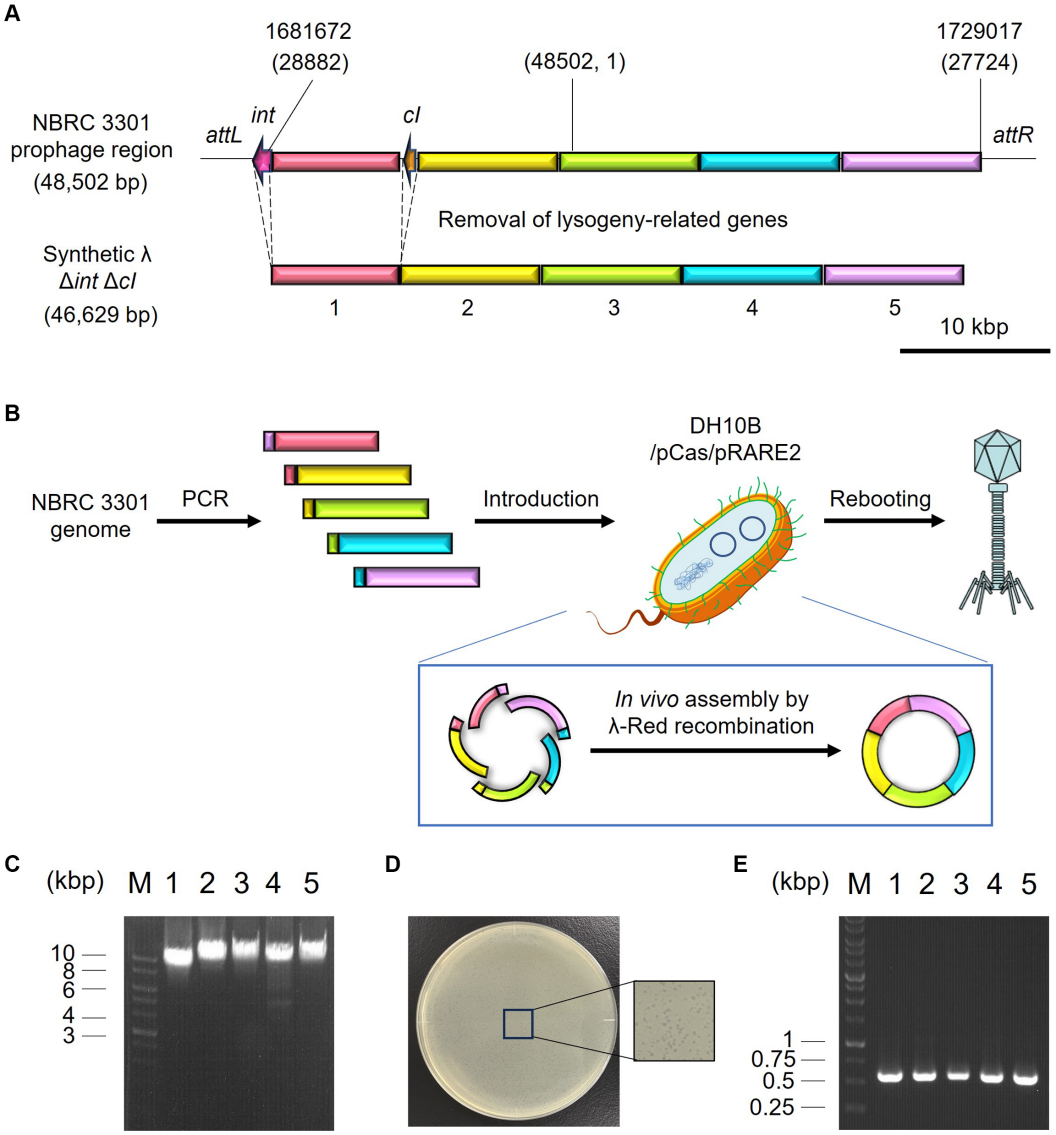
Synthesis of the artificial λ phage from the prophage region of a λ lysogen. **(A)** Design of the virulent derivative of the λ phage. Five regions except for *attL*, *int*, *cI*, and *attR* genes were amplified from the prophage region of the *E. coli* NBRC 3301 genome and assembled. The numbers at the top of the panel show the location in the NBRC 3301 genome (Genbank, BJLE01000002.1), while the numbers in parentheses show the location in the λ phage (GenBank, J02459.1). **(B)** Schematic illustration of *in vivo* DNA assembly and rebooting of the synthetic λ phage. **(C)** Results of agarose gel electrophoresis of the five λ fragments amplified by PCR. M, marker; 1–5, λ fragments 1 to 5. **(D)** Results of the plaque assay using the synthetic virulent λ. **(E)** Confirmation of gene assembly by amplifying the 600-bp joint regions of each fragment. M, marker; 1–5, joint regions of fragments 1 & 2, 2 & 3, 3 & 4, 4 & 5, and 5 & 1, respectively.

Microbiome engineering by natural phages involves the time-consuming and labor-intensive process of phage isolation. In addition, the current phage-isolation technique based on the plaque assay is not applicable to unculturable bacteria. If the lytic phages can be synthesized from prophage in lysogenic strains, phages infecting target bacterium can rapidly be obtained without cultivating target strain. Thus, the use of artificial phages allows microbiome engineering to become more widely available. Accordingly, the synthetic λ Δ*int* Δ*cI* phage was constructed, as illustrated in Figures 5A,B, and a test was carried out to determine whether the artificial phage and the natural phages can be used for microbiome engineering similar to the natural phages. Initially, *in vivo* DNA assembly and rebooting were performed using eight prophage DNA fragments, but rebooting was not possible. Therefore, the number of phage DNA fragments was reduced from eight to five to improve the efficiency of DNA assembly. Agarose gel electrophoresis analysis confirmed the amplification of each of the five λ phage-derived DNA fragments (8,402, 10,014, 9,552, 9,550, and 9,808 bp, respectively), as shown in Figure 5C. Through *in vivo* DNA assembly and phage rebooting, the synthetic λ phage was successfully constructed, as many small plaques were formed on the soft agar medium (Figure 5D). A single plaque was selected and 600 bp of the joining regions of the assembled DNA fragments were amplified by PCR directly from the plaque. As anticipated, DNA bands corresponding to the expected size of 600 bp were detected in the agarose gel electrophoresis analysis (Figure 5E). Further sequencing analyses revealed that the joining regions had the expected sequences (Supplementary Table S2), suggesting that the five DNA fragments derived from the prophage in the λ lysogen were correctly assembled.

Prior to microbiome engineering, the dose dependency of λ Δ*int* Δ*cI* was evaluated. Although the growth of *E. coli* Cm^R^ after 10 h of cultivation was inhibited by phage treatment at both MOI values of 0.001 and 0.01, the viable cell counts (3.11 × 10^5^ CFU/mL and 3.29 × 10^5^ CFU/mL, respectively) were higher than the initial cell concentration of 1.00 × 10^5^ CFU/mL (Supplementary Figure S2). On the other hand, the addition of 10^4^ PFU/mL (MOI = 0.1) of λ Δ*int* Δ*cI* was enough to inhibit the growth of *E. coli*. No reduction of the viable cell counts was observed between the culture with and without the addition of λ Δ*int* Δ*cI* in the nonhost bacteria: 3.42 × 10^9^ CFU/mL and 1.13 × 10^9^ CFU/mL for *P. putida* Neo^R^ (*p* = 0.24), 9.60 × 10^7^ CFU/mL and 4.80 × 10^7^ CFU/mL for *B. subtilis* Str^R^ (*p* = 0.085), and 9.20 × 10^6^ CFU/mL and 7.53 × 10^6^ CFU/mL for *L. plantarum* (*p* = 0.26), respectively (Supplementary Figure S3). These results indicated that similar to the T7 phage, λ Δ*int* Δ*cI* is highly specific for its host *E. coli*.

**Figure 6:**
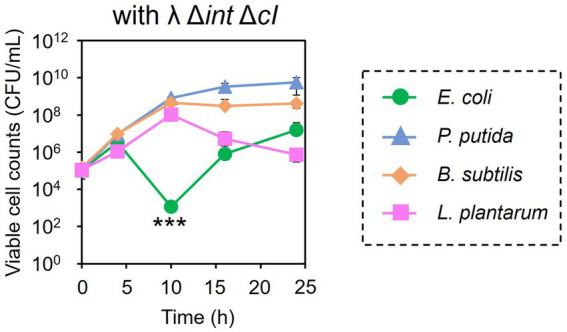
Microbiome engineering of the artificial microbiome by using the synthetic λ Δ*int* Δ*cI*. *E. coli* (green circles), *P. putida* (blue triangles), *B. subtilis* (brown diamonds), and *L. plantarum* (pink squares) were cocultivated for 24 h with λ Δ*int* Δ*cI* at MOI of 0.1. The samples were collected at 0, 4, 10, 16, and 24 h, and viable cell numbers were determined. Data dots show the mean ± standard deviation of three independent experiments. For statistical analyses, the viable cell counts of *E. coli* Cm^R^ were compared with its initial cell concentration. Triple asterisks indicate *p* values less than 0.005 in the *t*-test.

Encouraged by these results, the subtractive modification of an artificial microbiome by λ Δ*int* Δ*cI* was performed (Figure 6). The viable cell count of *E. coli* Cm^R^ was reduced to 1.17 × 10^3^ CFU/mL at 10 h by treatment with λ Δ*int* Δ*cI*. However, no reduction was observed in the viable cell counts of *P. putida*, *B. subtilis*, and *L. plantarum* (8.20 × 10^8^, 4.60 × 10^8^, and 1.04 × 10^8^ CFU/mL at 10 h, respectively). These results successfully demonstrated that microbiome engineering can be possible by using synthetic virulent phages rebooted from prophage DNA from a lysogenic strain.

## 4 Discussion

Bacteriophages have been widely used in the field of phage therapy, which aims to treat infections caused by pathogenic bacteria, including their antibiotic-resistant mutants. Meanwhile recent studies have emphasized the importance of maintaining a healthy gut microbiome (Manor et al., 2020), and bacteriophages are also attracting attention as a tool for precise microbiome engineering (Hsu et al., 2020; Ping et al., 2022). Although phages are believed to be specific at the genus, species, and sometimes strain levels (Nale et al., 2016; Gao et al., 2018), previous studies have evaluated their specificity by individually checking their lytic activity against nontarget bacteria in pure culture systems, not in a complex culture system (Nale et al., 2016; Gao et al., 2018; Fujiki et al., 2022). Therefore, in this study, we constructed an artificial microbiome consisting of four bacteria that do not inhibit the growth of one another (Supplementary Figure S4) and evaluated the host specificity of bacteriophages in this complex culture systems. Our results clearly showed that phages, whose specificity was confirmed in monoculture systems, maintained their specificity in the complex culture system and could reduce the numbers of their target bacterium without affecting nontarget bacteria (Figures 3, 4). Some studies have reported that the addition of phages to a microbiome altered the populations of nontarget bacteria, and the authors insisted that this was caused by a loss of interactions with target bacteria, not by nonspecific lysis by phages (Febvre et al., 2019; Hsu et al., 2019; Ping et al., 2022). In these studies, the host specificity of phages was confirmed only in pure culture systems, but our finding provides reason to believe that these phages are host-specific even in complex culture systems. Taken together, phages can be used for specifically reducing the numbers of a target bacterium when it does not interact with other bacteria, as well as for detecting its interactions with nontarget bacteria when such interactions are present. In the latter case, if target bacterium promote/inhibit the growth of other bacteria, the death of target bacterium will lead to the inhibition/promotion of the growth of other bacteria. Such experiments will reveal growth linkages among bacteria that make up microbiome. However, this will be possible only when phages do not induce cascading effects. Unlike the artificial microbiome used in this study, there are the myriad of interactions in real microbiome. If a microorganism, whose growth links to a target bacterium, has growth linkage with another microorganism, the addition of a phage infecting the target bacterium will cause change in the population of both bacteria by cascading effect. As a result, it becomes difficult to determine which bacteria have growth linkage with the target microorganism. However, this problem will be solved by cocultivating only those microorganisms that appear to have growth linkage *in vitro*. Therefore, to analyze growth linkages among bacteria in complex natural microbiomes, it is important to combine subtractive approach with a bottom-up approach.

One of the most important findings of this study is that the artificial microbiome could undergo a subtractive modification using natural phages, as well as the artificial virulent phage, which is synthesized from prophage DNA from a lysogenic strain (Figure 6). Phage-based microbiome engineering assumes that phages are available (Hsu et al., 2019). However, it is time-consuming and labor-intensive to isolate phages from environmental samples, in which it is not even known whether phages with the desired host range are present. In addition, the current method for phage screening depends on the plaque assay using purely cultured strains, not being applicable to uncultured bacteria. Conversely, recent advances in synthetic biology have enabled the creation of artificial phages (Kilcher et al., 2018; Mageeney et al., 2020; Cheng et al., 2022; Mitsunaka et al., 2022). Remarkably, the “virulent conversion” of a temperate phage was achieved by synthesizing and rebooting an artificial phage genome, in which the gene cluster encoding the lysogeny control function was removed (Mageeney et al., 2020; Cheng et al., 2022). Although in their study the artificial phages were synthesized from the genomes of the isolated λ phage and the phage infecting *Pseudomonas aeruginosa*, we succeeded in synthesizing the virulent mutant of λ phage from prophage DNA in the λ lysogen. This result indicates that virulent phages could be synthesized without isolating phages if prophage sequences of target species are available. By adding synthetic phages to microbiomes, it would be possible to target microorganisms, including uncultured ones, in microbiomes.

The key obstacles to implementing this strategy in real complex microbiomes are (1) accurately determining prophage sequences from metagenomic sequences and (2) artificially synthesizing diverse phages. There are several tools to predict prophage sequences from genome sequences such as PHASTER (Arndt et al., 2016), PhageBoost (Sirén et al., 2021), and DEPhT (Gauthier et al., 2022). However, prediction results vary among tools (Gauthier et al., 2022), and it is difficult to distinguish intact prophages from defective or cryptic ones. As a reliable method for predicting the prophage sequences, Fujimoto et al. (2020) performed a comparative analysis of phage sequences with genomic sequences of intestinal microorganisms. When prophages enter the lytic cycle, phages infect host cells and replicate their DNA via a circular intermediate. Therefore, by finding sequences in the bacterial genomes that are identical to circular viral contigs, the sequences of active prophages can be determined. These prophage sequences could be used as blueprints for synthesizing artificial virulent phages for microbiome engineering. Importantly, the virulent conversion of a prophage was performed by DNA rebooting in this study. While virulent conversion can also be possible by removing the lysogeny control region of the prophage in the host genome by genome engineering and following phage induction (Tinoco et al., 2016; Jin et al., 2022), genome engineering is applicable to only a few microorganisms. On the contrary, phage rebooting is based on the assembly of phage DNA fragments and following rebooting of synthetic phages in non-native host, and will be applicable for a wide variety of phages. The rationale for this assumption is that the host specificity of phages largely arises from the affinity between the tail extremity of phages and receptors on the cell surface of the host bacterium (Klumpp et al., 2023). Therefore, if phage DNA can be introduced into bacterial cells, it will be possible to reboot phages across species and genera in a single host strain specialized for phage rebooting. In fact, Cheng et al. (2022) succeeded in rebooting phages infecting Gram-negative bacteria such as *Klebsiella pneumoniae*, *Salmonella enterica*, *P. aeruginosa*, and *Acinetobacter baumannii* by introducing their respective phage genome into *E. coli* as a rebooting host. However, it will be difficult to reboot all phages using a single host strain because phage promoter must be recognized by host RNA polymerase to initiate by transcription. One strategy to overcome this problem is to expand rebooting host. Kilcher et al. (2018) reported that the phages infecting Gram-positive bacteria such as *Bacillus cereus*, *Bacillus thuringiensis*, *Listeria ivanovii*, and *Staphylococcus aureus* could be rebooted using *Listeria monocytogenes* as a rebooting host. Another strategy is to make *E. coli* recognize diverse phage promoters. Since the sigma factor subunits of *E. coli* RNA polymerase can be replaced with other sigma factors to recognize heterologous promoters (Gaida et al., 2015; Emslander et al., 2022), it may be possible to make *E. coli* reboot more diverse phages by co-expressing heterologous sigma factor in addition to λ-Red recombinase. These improvements will thus increase the convenience of phage-based microbiome engineering. Interestingly, combination of *in vitro* DNA assembly techniques such as Gibson assembly and cell-free phage synthesis system allowed the construction of artificial bacteriophage *in vitro* (Levrier et al., 2024). Therefore, the use of cell-free system will be another option for artificially synthesizing diverse phages.

Synthetic strategy of bacteriophages will also solve the problems found in microbiome engineering in this study. The bacteriolytic activity of ΦLpTT2 was weaker than those of T7, ΦPpMK2-1, and ΦBsKO1-1 (Figure 1). ΦLpTT2 did not reduce the number of *L. plantarum* and only inhibited its growth (Figure 3E). In addition, while both T7 phage and λ Δ*int* Δ*cI* could reduce the viable cell numbers of *E. coli* in microbiome engineering experiments (Figures 3B, 6), λ Δ*int* Δ*cI* showed lower lytic ability (Figure 1 vs. Supplementary Figure S2). Such differences in infection capability are probably due to differences in various factors in phage infection such as adsorption rate to the host cells, latent period, phage productivity, lysis time, and burst size (Kannoly et al., 2022). As a method to enhance the killing ability of phages, Jin et al. (2022) synthesized an engineered λ phage containing a CRISPR-Cas3 system. The CRISPR-Cas3 system was programmed to target and degrade the host genome, and the synthetic λ phage showed higher *E. coli* killing activity than that of the wild-type λ phage. Phage engineering thus makes it possible to synthesize phages with high host-killing activity without relying on phage screening. Another problem is the occurrence of the phage-resistant mutants. During microbiome engineering using the phages, we observed growth restoration, especially in *E. coli* and *P. putida* (Figures 3B,C). It has been reported that phage-resistant strains rapidly emerge when host bacteria are in continuous contact with the phages (Loc Carrillo et al., 2005; Oechslin et al., 2017; Hesse et al., 2020), suggesting that the observed growth restoration in our study was due to the emergence of phage-resistant strains. In fact, the two *E. coli* and *P. putida* strains recovered from the colonies formed after 24 h of microbiome modification experiments (Figures 3B,C) showed no or low susceptibility to T7 and ΦPpMK2-1 phages, respectively (Supplementary Figure S5). The alteration of surface structure caused by DNA mutations is one of the main phage resistance mechanisms of bacteria (McGee et al., 2023). Since phages with different host ranges recognize different receptors on the cell surface of host bacteria (Yehl et al., 2019), combined use of phages recognizing different receptors as a phage cocktail allows phage infection by different mechanisms. Therefore, bacteria can grow only when they develop phage resistance against all phages in phage cocktail (Hesse et al., 2020). As a result, the use of phage cocktails can delay the emergence of resistant bacteria (Smug et al., 2022). However, the isolation of phages recognizing different receptors on the same host is laborious. Yehl et al. (2019) randomized the host-range-determining regions in the tail fiber protein of the T3 phage and selected mutant phages that can infect T3-resistant strains. Meanwhile, Ando et al. (2015) synthesized the artificial phages with altered host ranges by swapping tail fibers genes between different phages. These strategies will allow the rapid development of phage cocktails and prolong the emergence of phage-resistant strains.

In conclusion, we demonstrated that bacteriophages can specifically reduce the numbers of their host bacterium without perturbing the growth of nonhost bacteria, thus enabling them to be used for precise microbiome engineering. Microbiome engineering was also possible by using the artificial virulent phage synthesized from the prophage DNA of a lysogenic strain. Therefore, the synthesis of “tailor-made” phages using prophage DNAs in metagenomic sequences as blueprints and following microbiome engineering would be possible. Advances in phage synthesis and microbiome engineering will accelerate the functional analysis of microorganisms of interest in microbiomes, along with the creation of synthetic microbiomes with desired functions. The elucidation of the health functions of gut microorganisms, the discovery of key microorganisms responsible for nutrients cycling to increase plant productivity, and the discovery of key degraders of harmful substances in the soil are the potential applications.

## Data availability statement

The original contributions presented in the study are included in the article/Supplementary material, further inquiries can be directed to the corresponding author. The genome sequences of ΦLpTT2, ΦPpMK2-1, and ΦBsKO1-1 are available from DDBJ/EMBL/GenBank with the accession numbers LC805022, LC805023, and LC805024. The associated BioProject, BioSample, and Sequence Read Archive accession numbers are PRJDB17660/SAMD00754419/DRR536257, PRJDB17658/SAMD00754420/DRR536258, and PRJDB17659/SAMD00754421/DRR536259, respectively.

## Author contributions

TT: Formal analysis, Investigation, Methodology, Writing – original draft. RS: Investigation, Methodology, Writing – review & editing. YS: Formal analysis, Investigation, Writing – review & editing. MK: Investigation, Writing – review & editing. KH: Supervision, Writing – review & editing. HI: Supervision, Writing – review & editing. KO: Conceptualization, Formal analysis, Funding acquisition, Investigation, Methodology, Project administration, Writing – original draft.

## Footnotes

^1^ http://n2t.net/addgene:62225
