# Supplementary material for: Precise microbiome engineering using natural and synthetic bacteriophages targeting an artificial bacterial consortium
Source: Front Microbiol. 2024 May 2;15:1403903. doi: 10.3389/fmicb.2024.1403903 (PMC11096457; doi:10.3389/fmicb.2024.1403903)
Supplement: Supplementary file 1 [file Data_Sheet_1.pdf]

Supplementary Material

Precise Microbiome Engineering Using Natural and Synthetic Bacteriophages Targeting an Artificial Bacterial Consortium

Tomoki Tanaka[1], Ryoga Sugiyama[1], Yu Sato[2], Manami Kawaguchi[3], Kohsuke Honda[4, 5], Hiroaki Iwaki[3], Kenji Okano[3]*

[1]Department of Chemistry, Materials and Bioengineering, Graduate School of Science and Engineering, Kansai University, Osaka, Japan

[2]Division of Life Science, Graduate School of Sciences and Technology for Innovation, Yamaguchi University, Yamaguchi, Japan

[3]Department of Life Science and Biotechnology, Faculty of Chemistry, Materials and Bioengineering, Kansai University, Osaka, Japan

[4]International Center for Biotechnology, Osaka University, Osaka, Japan

[5]Industrial Biotechnology Initiative Division, Institute for Open and Transdisciplinary Research Initiatives, Osaka University, Osaka, Japan

*** Correspondence:**
Kenji Okano
okano.k@kansai-u.ac.jp

1 Supplementary Materials and Methods

1.1 Extraction of the phage genomes

For amplification of ΦPpMK2-1, ΦBsKO1-1, and ΦLpTT2, the plaque assay was performed using the phage suspensions diluted at various dilution rates. SM buffer was added to the agar plate, where nearly confluent plaques were formed on the top agar, and phages were extracted from the top agar by crushing it using a spreader and suspending agar pieces in SM buffer. The supernatant was collected and impurities were removed by centrifugation at 6,000 × *g* for 5 min, followed by filtration through a 0.22 µm filter (PES025022S; Membrane Solutions, Auburn, WA, USA). To eliminate host-derived DNA, 10 µL of DNase I (Norgen Biotek Corp., Ontario, Canada) was added to the 1 mL of phage suspensions (> 10^{10} PFU/mL), followed by incubation at room temperature for 15 min. DNase I was then inactivated by adding 10 µL of 0.5 M EDTA solution (pH = 8.0) and incubated at 75°C for 5 min. After these treatments, the phage genomes were extracted using a Phage DNA Isolation Kit (Norgen Biotek Corp.) according to the manufacturer's instructions.

1.2 Whole-genome sequencing analysis

Whole-genome sequencing analyses of the isolated phages were performed using the MinION Mk1C sequencer (Oxford Nanopore Technologies, Oxford, UK). DNA libraries were prepared using the Ligation Sequencing Kit V14 (LSK-SQ114; Oxford Nanopore Technologies) according to the manufacturer's instructions. DNA libraries were then applied to Flongle R10.4.1 Flow Cell on the sequencer and DNA sequencing was performed for 16 h.

The *de novo* assembly of the nanopore reads was performed according to the method described by Romero-Calle et al. (2023) with some modifications. From the nanopore reads, the adapter sequences were eliminated using the Porechop version 0.2.4 (**https://github.com/rrwick/Porechop**). Possible sequence errors in the sequences were corrected using the Canu version 2.2 (Koren et al., 2017). Flye version 2.9.3 was utilized for the *de novo* assembly of the trimmed and corrected reads (Kolmogorov et al., 2019). Racon version 1.4.17 (https://github.com/isovic/racon) was used to obtain consensus sequences using the mapped data with minimap2 version 2.26-r1175 (Li, 2018). To remove sequences derived from the host, the phages detected in all three programs, Virsorter2 (Guo et al., 2021), geNomad (Camargo et al., 2023), and DeepVirFinder (Ren et al., 2020), were used for further analyses. geNomad was also utilized to determine the number of genes in ΦPpMK2-1, ΦBsKO1-1, and ΦLpTT2. Phylogenetic analyses were performed using Viptree version 4.0 (Nishimura et al., 2017).

2 Supplementary Figures and Tables

2.1 Supplementary Figures

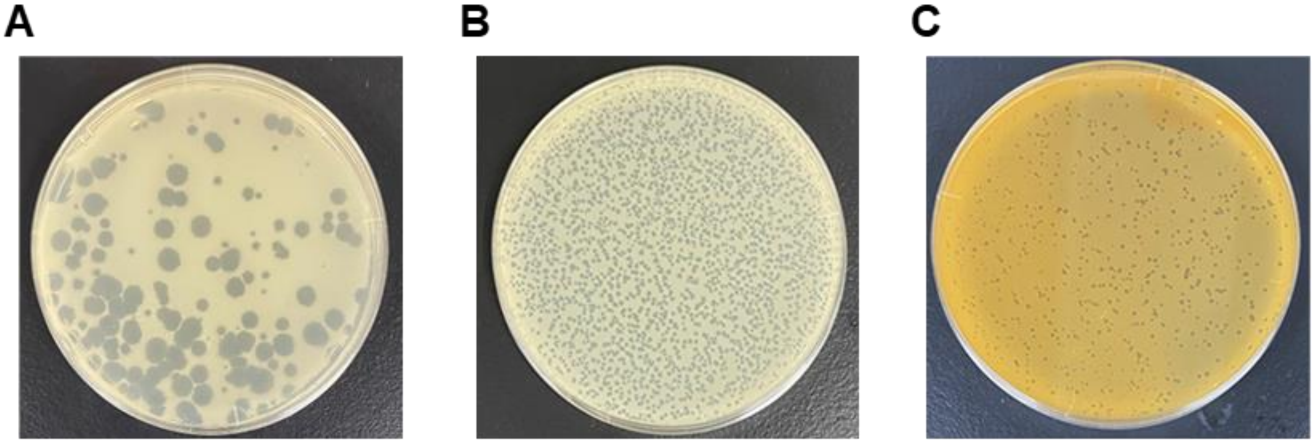

Supplementary Figure 1. Isolation of the bacteriophages from the soil extract and wastewater. The concentrated soil extract and wastewater were filtered through a 0.22 µm pore-size syringe filter and mixed with cultures of *P. putida*, *B. subtilis*, or *L. plantarum*. The mixtures were added to 0.5% (w/v) soft agar and overlaid to 1.5% (w/v) agar media. After overnight cultivation, plaque formation was observed. The photos show the plaques formed on the lawn of (A) *P. putida*, (B) *B. subtilis*, and (C) *L. plantarum*.

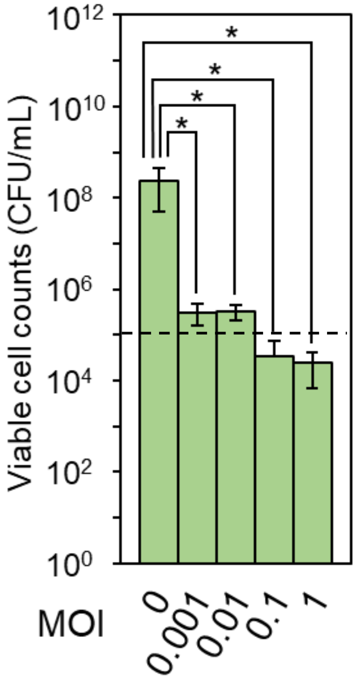

Supplementary Figure 2. Examination of the dose dependency of λ Δ*int* Δ*cI* for inhibiting the growth of *E. coli*. λ Δ*int* Δ*cI* was added to the culture of *E. coli* at different MOI values. After 10 h of cultivation, the viable cell counts of *E. coli* were measured. The dotted line represents the initial viable cell counts (1.00×10^5 CFU/mL). Data bars show the mean ± standard deviation of three independent experiments. The viable cell counts of *E. coli* with and without λ Δ*int* Δ*cI* were compared. Asterisks indicate *p* values less than 0.05 in the *t*-test.

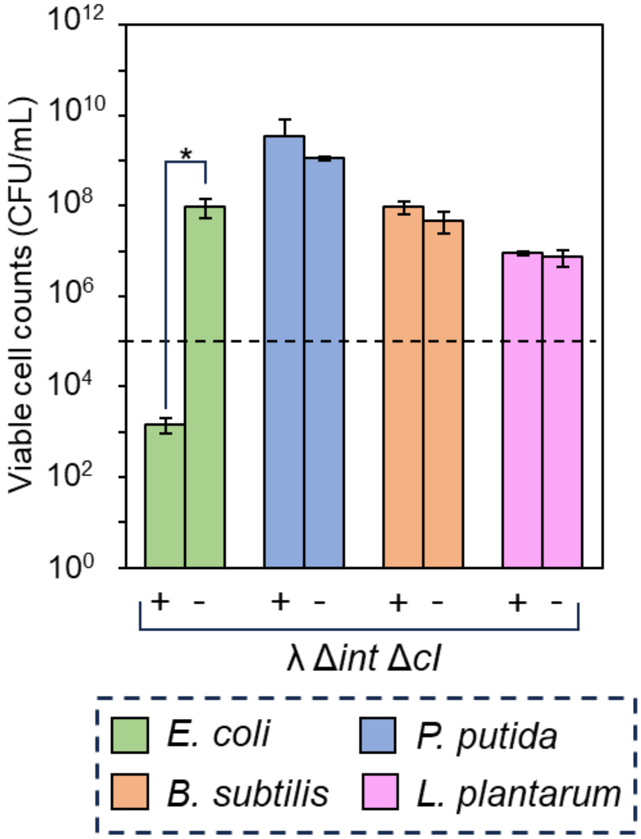

Supplementary Figure 3. Evaluation of the host specificity of λ Δ*int* Δ*cI*. λ Δ*int* Δ*cI* was added to the culture of *E. coli*, *P. putida*, *B. subtilis*, and *L. plantarum* at MOI of 0.1. The dotted line represents the initial viable cell counts (1.00×10^5 CFU/mL). Data bars show the mean ± standard deviation of three independent experiments. For statistical analyses, the viable cell counts of each bacterium with and without the addition of λ Δ*int* Δ*cI* were compared. Asterisks indicate *p* values less than 0.05 in the *t*-test.

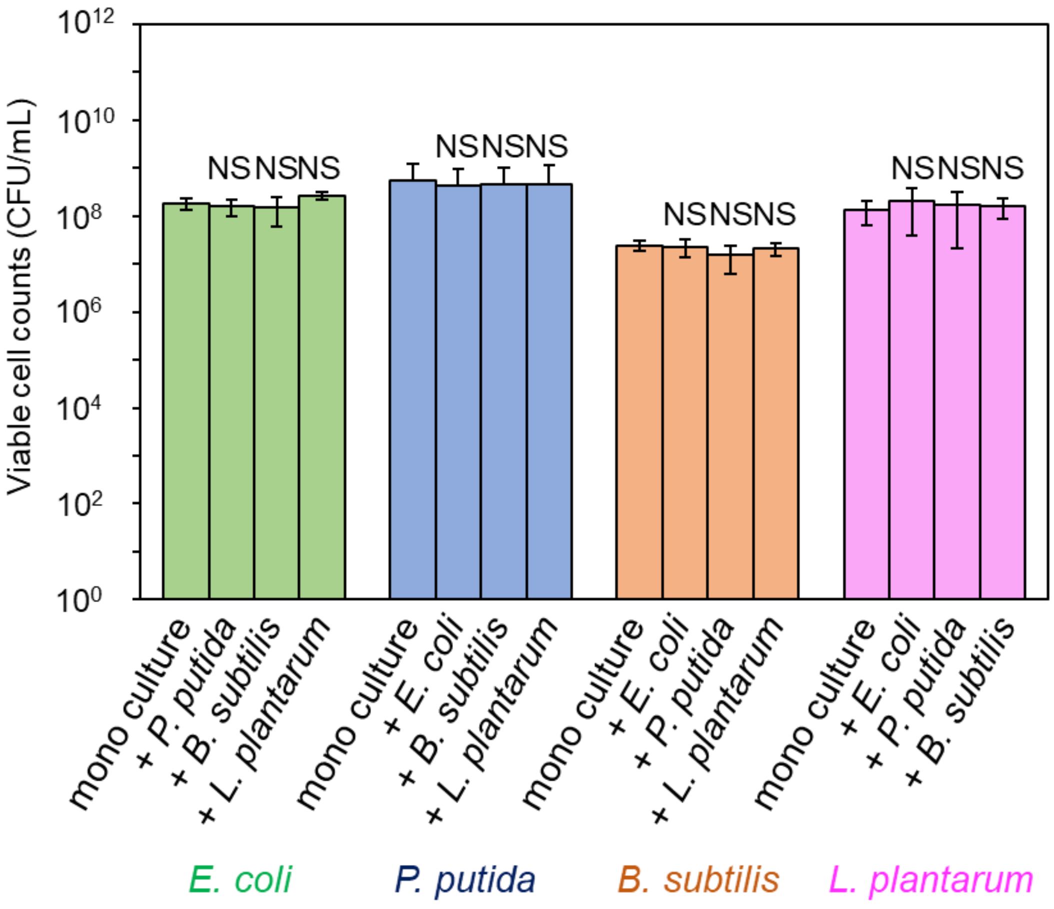

Supplementary Figure 4. Comparison of bacterial growth between monoculture system and coculture systems. Each bacterium was cultivated without and with one of the other three bacteria. Data bars show the mean ± standard deviation of three independent experiments. For statistical analyses, the viable cell counts of each bacterium with and without the addition of one of the other bacteria were compared. NS indicates that there was no significant difference in the *t*-test.

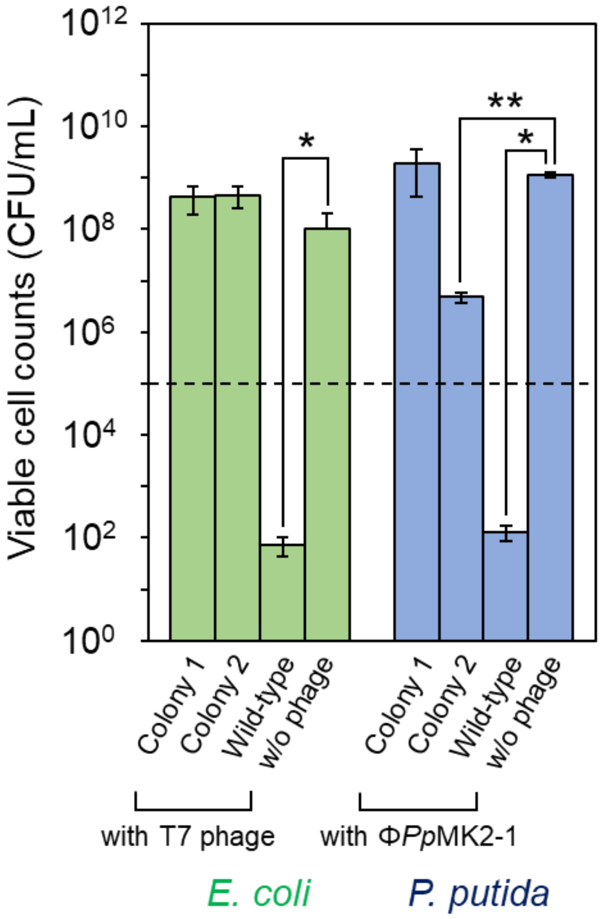

Supplementary Figure 5. Evaluation of susceptibility of the growth-restored strains to the phages. After 24 h of cultivation in the microbiome modification experiments (Figure 3), the viable cell numbers were counted. Then, two colonies of *E. coli* and *P. putida* were picked up and their susceptibility to the phages were compared with that of their parental strain. T7 phage (MOI 0.001) and ΦPpMK2-1 (MOI 0.01) was added in cultures of *E. coli* and *P. putida*, respectively. The dotted line represents the initial viable cell counts (1.00×10^5 CFU/mL). Data bars show the mean ± standard deviation of three independent experiments. For statistical analyses, the viable cell counts of each bacterium with and without the addition of the bacteriophage were compared. Asterisks and double asterisks indicate *p* values less than 0.05 and 0.01 in the *t*-test, respectively.

2.2 Supplementary Tables

Supplementary Table 1. Sequences of the oligonucleotide primers used for the construction of the synthetic λ phage

Primer	Sequence (5'-3')	Amplified DNA
For DNA assembly		
λ 28883-28907_F	TATATCATTTTACGTTTCTCGTTCATTCTGATCCTCTTCAAAAGGCCACC	λ fragment 1
λ 37207-37226_R	GATATTTATCCCTTGCGGTGATAGATTTAACGTTCGGCAAGGTGTTCTGGTCG	
λ 37941-37980_F	GAACACCTTGCCGAACGTTAAATCTATCACCGCAAGGGATAAATATCTAACACC	λ fragment 2
λ 47450-47490_R	AAACTACGCGCCCTCGTATCACATGGAAGGTTTTACCAATG	
λ 47441-47475_F	AACCTGAGCCATTGGTAAAACCTTCCATGTGATAC	λ fragment 3
λ 8457-8490_R	CACGCCGTATCCGCTCAATATTTTGTTTAAACGC	
λ 8441-8480_F	GGTGCCGCTGACCACGGCGTTTAAAC	λ fragment 4
λ 17962-17990_R	TTGCCGAGATGGGATTCGGTTATCTTGCC	
λ 17941-17988_F	GGTTACCTGGATTTTTTCAAAGGCAAGATAACCGAATCCCATCTCGGC	λ fragment 5
λ 27693-27723_R	GGTGGCCTTTTGAAGAGGATCAGAATGAACGAGAAACGTAAAATGATATAAATATC	
For confirmation of assembly		
λ 36927-36957_F	CTCTTGTCATTTGTTTTGGTAAAGAGAAAAG	Joint region of λ fragment 1 & 2
λ 38215-38240_R	TATGCTGTTGTTTTTTTGTTACTCGG	
λ 47191-47218_F	ATGTGATTTCTCTTGATTTCAACCTATC	Joint region of λ fragment 2 & 3
λ 47769-47790_R	TTTACACGAATCAGATCCACGG	
λ 8191-8211_F	GAATCAAAGTTAACCGGGGGG	Joint region of λ fragment 3 & 4
λ 8771-8790_R	CACGCATCCAGCTCTGAATC	
λ 17691-17710_F	AACCGCCACGCCGCATCTTG	Joint region of λ fragment 4 & 5
λ 18261-18290_R	AACACGTCGTTCATGAATATCTGGTTGCCC	
λ 27424-27449_F	TTGACATCACTGCTATCTTCTTACTG	Joint region of λ fragment 5 & 1
λ 29161-29182_R	AGATAGCACATGCAGACGTAAC	

Supplementary Table 2. Sequencing analyses of the joint regions of the assembled DNA fragments in λ Δ*int* Δ*cI*

Joining region [a]	Sequence (5'-3')
Fragments 1 & 2<br>(36965-37226,<br>37941-38227)	ATATACAAATAATTGGAGCCAACCTGCAGGTGATGATTATCAGCCAGCAGAGAATTAAGGAAAACAGACAGGT<br>TTATTGAGCGCTTATCTTTCCCTTTATTTTTGCTGCGGTAAGTCGCATAAAAACCATTCTTCATAATTCAATCCAT<br>TTACTATGTTATGTTCTGAGGGGAGTGAAAATTCCCCTAATTCGATGAAGATTCTTGCTCAATTGTTATCAGCTA<br>TGCGCCGACCAGAACACCTTGCCGAACGTTAAATCTATCACCGCAAGGGATAAATATCTAACACCGTGCGTGTT<br>GACTATTTTACCTCTGGCGGTGATAATGGTTGCATGTACTAAGGAGGTTGTATGGAACAACGCATAACCCTGAA<br>AGATTATGCAATGCGCTTTGGGCAAACCAAGACAGCTAAAGATCTCGGCGTATATCAAAGCGCGATCAACAAG<br>GCCATTCATGCAGGCCGAAAGATTTTTTAACTATAAACGCTGATGGAAGCGTTTATGCGGAAGAGGTAAAGCC<br>CTTCCCGAGTAACAAAA
Fragments 2 & 3<br>(47237-47771)	ATGCGTGTTTCTTGAGAATTTAACATTTACAACCTTTTTAAGTCCTTTTATTAACACGGTGTTATCGTTTTCTAAC<br>ACGATGTGAATATTATCTGTGGCTAGATAGTAAATATAATGTGAGACGTTGTGACGTTTTAGTTCAGAATAAAA<br>CAATTCACAGTCTAAATCTTTTCGCACTTGATCGAATATTTCTTTAAAAATGGCAACCTGAGCCATTGGTAAAAC<br>CTTCCATGTGATACGAGGGCGCGTAGTTTGCATTATCGTTTTTATCGTTTCAATCTGGTCTGACCTCCTTGTGTTT<br>TGTTGATGATTTATGTCAAATATTAGGAATGTTTTCACTTAATAGTATTGGTTGCGTAACAAAGTGCGGTCCTGC<br>TGGCATTCTGGAGGGAAATACAACCGACAGATGTATGTAAGGCCAACGTGCTCAAATCTTCATACAGAAAGAT<br>TTGAAGTAATATTTTAACCGCTAGATGAAGAGCAAGCGCATGGAGCGACAAAATGAATAAAGAACAATCTGCT<br>GATGATCCCTCCG
Fragments 3 & 4<br>(8236-8756)	ATGCGCGGGTTGTCCTTTCGCGCCGCAGGCGTCGTAAAAAGGGGCAGCGTTCATCCCTGAAAGGTGGCGGCAG<br>CGTGCTTGTGGTGGGTAACCGTCGTATTCCCGGCGCGTTTATTCAGCAACTGAAAAATGGCCGGTGGCATGTCA<br>TGCAGCGTGTGGCTGGGAAAAAACCGTTACCCCATTGATGTGGTGAAAATCCCGATGGCGGTGCCGCTGACCA<br>CGGCGTTTAAACAAAATATTGAGCGGATACGGCGTGAACGTCTTCCGAAAGAGCTGGGCTATGCGCTGCAGCA<br>TCAACTGAGGATGGTAATAAAGCGATGAAACATACTGAACTCCGTGCAGCCGTACTGGATGCACTGGAGAAGC<br>ATGACACCGGGGCGACGTTTTTTGATGGTCGCCCCGCTGTTTTTGATGAGGCGGATTTTCCGGCAGTTGCCGTTT<br>ATCTCACCGGCGCTGAATACACGGGCGAAGAGCTGGACAGCGATACCTGGCAGGCGGAGCTGCATATCGAAGT<br>TTTCCTGC
Fragments 4 & 5<br>(17733-18266)	GTTTGAGTTCTGGTTCTCGGAAAAGCAGATTGCGGATATCAGACAGGTTGAAACCAGCACGCGTTATCTTGGTA<br>CGGCGCTGTACTGGATAGCCGCCAGTATCAATATCAAACCGGGCCATGATTATTACTTTTATATCCGCAGTGTG<br>AACACCGTTGGCAAATCGGCATTCGTGGAGGCCGTCGGTCGGGCGAGCGATGATGCGGAAGGTTACCTGGATT<br>TTTTCAAAGGCAAGATAACCGAATCCCATCTCGGCAAGGAGCTGCTGGAAAAAGTCGAGCTGACGGAGGATAA<br>CGCCAGCAGACTGGAGGAGTTTTCGAAAGAGTGGAAGGATGCCAGTGATAAGTGGAATGCCATGTGGGCTGTC<br>AAAATTGAGCAGACCAAAGACGGCAAACATTATGTCGCGGGTATTGGCCTCAGCATGGAGGACACGGAGGAA<br>GGCAAACTGAGCCAGTTTCTGGTTGCCGCCAATCGTATCGCATTTATTGACCCGGCAAACGGGAATGAAACGCC<br>GATGTTTGTGGCGCAGGGCAA
Fragments 5 & 1<br>(27424-27723,<br>28883-29132)	TTGACATCACTGCTATCTTCTTACTGGTTATGCAGGTCGTAGTGGGTGGCACACAAAGCTTTGCACTGGATTGCG<br>AGGCTTTGTGCTTCTCTGGAGTGCGACAGGTTTGATGACAAAAAATTAGCGCAAGAAGACAAAAATCACCTTGC<br>GCTAATGCTCTGTTACAGGTCACTAATACCATCTAAGTAGTTGATTCATAGTGACTGCATATGTTGTGTTTTACA<br>GTATTATGTAGTCTGTTTTTTATGCAAAATCTAATTTAATATATTGATATTTATATCATTTTACGTTTCTCGTTCA<br>TTCTGATCCTCTTCAAAAGGCCACCTGTTACTGGTCGATTTAAGTCAACCTTTACCGCTGATTCGTGGAACAGAT<br>ACTCTCTTCCATCCTTAACCGGAGGTGGGAATATCCTGCATTCCCGAACCCATCGACGAACTGTTTCAAGGCTTC<br>TTGGACGTCGCTGGCGTGCGTTCCACTCCTGAAGTGTCAAGTACATCGCAAAGTCTCCGCAATTACACGCAAGA<br>AAAAACCGCCATCAGGCGGCTTGGTG

[a] The numbers in parentheses show the location in the λ phage (GenBank, J02459.1). Removal of *cI* and *int* genes in λ Δ*int* Δ*cI* was confirmed by DNA sequencing of the joining regions of fragments 1 & 2 and fragment 5 & 1, respectively.

3 References

Camargo, A. P., Roux, S., Schulz, F., Babinski, M., Xu, Y., Hu, B. et al. (2023). Identification of mobile genetic elements with geNomad. *Nat. Biotechnol.*. doi: 10.1038/s41587-023-01953-y

Guo, J., Bolduc, B., Zayed, A. A., Varsani, A., Dominguez-Huerta, G., Delmont, T. O. et al. (2021). VirSorter2: a multi-classifier, expert-guided approach to detect diverse DNA and RNA viruses. *Microbiome* 9:37. doi: 10.1186/s40168-020-00990-y

Kolmogorov, M., Yuan, J., Lin, Y., and Pevzner, P. A. (2019). Assembly of long, error-prone reads using repeat graphs. *Nat. Biotechnol.* 37:540–546. doi: 10.1038/s41587-019-0072-8

Koren, S., Walenz, B. P., Berlin, K., Miller, J. R., Bergman, N. H., and Phillippy, A. M. (2017). Canu: scalable and accurate long-read assembly via adaptive k-mer weighting and repeat separation. *Genome Res.* 27:722–736. doi: 10.1101/gr.215087.116

Li, H. (2018). Minimap2: pairwise alignment for nucleotide sequences. *Bioinformatics* 34:3094–3100. doi: 10.1093/bioinformatics/bty191

Nishimura, Y., Yoshida, T., Kuronishi, M., Uehara, H., Ogata, H., and Goto, S. (2017). ViPTree: the viral proteomic tree server. *Bioinformatics* 33:2379–2380. doi: 10.1093/bioinformatics/btx157

Ren, J., Song, K., Deng, C., Ahlgren, N. A., Fuhrman, J. A., Li, Y. et al. (2020). Identifying viruses from metagenomic data using deep learning. *Quant. Biol.* 8:64–77. doi: 10.1007/s40484-019-0187-4

Romero-Calle, D. X., Pedrosa-Silva, F., Ribeiro Tomé, L. M., Fonseca, V., Guimarães Benevides, R., de Oliveira Santos, L. T. S. et al. (2023). Molecular characterization of Salmonella phage Wara isolated from river water in Brazil. *Microorganisms* 11:1837. doi: 10.3390/microorganisms11071837
